# Conical expansion of the outer subventricular zone and the role of neocortical folding in evolution and development

**DOI:** 10.3389/fnhum.2013.00424

**Published:** 2013-08-01

**Authors:** Eric Lewitus, Iva Kelava, Wieland B. Huttner

**Affiliations:** Max Planck Institute of Molecular Cell Biology and GeneticsDresden, Germany

**Keywords:** neocortex, gyrencephaly, subventricular zone, neural progenitors, mammals, extracellular matrix, phylogenetics

## Abstract

There is a basic rule to mammalian neocortical expansion: as it expands, so does it fold. The degree to which it folds, however, cannot strictly be attributed to its expansion. Across species, cortical volume does not keep pace with cortical surface area, but rather folds appear more rapidly than expected. As a result, larger brains quickly become disproportionately more convoluted than smaller brains. Both the absence (lissencephaly) and presence (gyrencephaly) of cortical folds is observed in all mammalian orders and, while there is likely some phylogenetic signature to the evolutionary appearance of gyri and sulci, there are undoubtedly universal trends to the acquisition of folds in an expanding neocortex. Whether these trends are governed by conical expansion of neocortical germinal zones, the distribution of cortical connectivity, or a combination of growth- and connectivity-driven forces remains an open question. But the importance of cortical folding for evolution of the uniquely mammalian neocortex, as well as for the incidence of neuropathologies in humans, is undisputed. In this hypothesis and theory article, we will summarize the development of cortical folds in the neocortex, consider the relative influence of growth- vs. connectivity-driven forces for the acquisition of cortical folds between and within species, assess the genetic, cell-biological, and mechanistic implications for neocortical expansion, and discuss the significance of these implications for human evolution, development, and disease. We will argue that evolutionary increases in the density of neuron production, achieved via maintenance of a basal proliferative niche in the neocortical germinal zones, drive the conical migration of neurons toward the cortical surface and ultimately lead to the establishment of cortical folds in large-brained mammal species.

## 1. Introduction

Cortical folding and brain development are tightly linked. The prenatal characterization of gyri and sulci may be used to identify functionally distinct cortical areas in many species and predict normal or pathological cerebral function at term. Close correlations between cortical morphology and behavioral function (or dysfunction) suggest that the early development of cortical folds constitutes an important step, either for normal development or as an indicator of normal development, in the construction of the human brain. Comparisons between normal and pathological human brains and between humans and other mammal species highlight important differences in progenitor cell-type abundances, cell-cycle dynamics, radial fiber organization, and gene expression profiles that account for gross phenotypic differences in neocortical morphology and function and even organismal behavior (Bayer and Altman, 1991; Beaulieu, 1993; Dehay et al., 1993; Polleux et al., 1997a,b; Lukaszewicz et al., 2005; Dubois et al., 2008; Toro et al., 2008; Clowry et al., 2010; Fietz et al., 2010, 2012; Hansen et al., 2010; Zilles et al., 2013).

## 2. The chronology of neocortical folding during development is highly regulated and conserved across species

**Figure 1:**
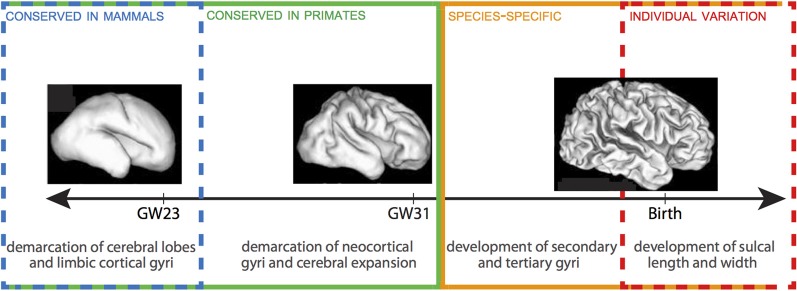
**Timeline of gyrification in human.** Stages 1 and 2 are delineated by GW 31-32. There is a progressive lack of conservation in cortical folding patterns toward the final stages of gyrification, as minor developmental changes in gyri and sulci become increasingly specialized to species and, ultimately, susceptible to local environmental and experiential variations. 3D reconstructions of fetal human brains from Barnette et al. (2009). Figure follows Sawada et al. (2012b).

The emergence of neocortical gyri and sulci can be summarized in two stages: (1) the demarcation of primary gyri at human gestation weeks (GW) 23–31; and (2) the emergence of secondary gyri and the growth of sulcal length and depth between late stages of fetal development and early stages of postnatal life (Figure 1) (Chi et al., 1977; Armstrong et al., 1995; Mayhew et al., 1996). Stage 1, which follows the demarcation of cerebral lobes and limbic cortical gyri, is largely conserved between humans and other gyrencephalic primates. The correlative increase in cerebral volume and gyrification during this stage, including a dramatic increase in gyri in the occipital region, may in fact constitute the formation of a characteristic pattern of gyrencephaly common to all gyrencephalic primates. Work in Old World monkeys has shown that all neocortical gyri, with the exception of the superior temporal gyrus, emerge during Stage 1 and that both the chronology of emergence and rostrocaudal distribution of gyri are homologous in monkeys and humans (Zilles et al., 1988; Rilling and Insel, 1999; Sawada et al., 2012a,b). There is, despite this broad conservation, a delayed emergence of the parietoccipital gyri (e.g., cuneus, angular gyrus, supramarginal gyrus) in humans compared to monkeys, which, because these gyri are associated with Wernicke's area in humans but dorsal extrastriate cortex in monkeys (Sawada et al., 2012a,b), may indicate that heterochronic changes in gyri emergence reflect species-specific adaptations in particular cortical regions.

Across all mammal species, cortical folds accumulate non-linearly with increasing brain volume, such that, per gram, larger brains are more gyrencephalic than smaller brains (Zilles et al., 2013). Within species, gyrencephaly index (GI) shows high levels of heritability, but is negatively correlated with both cerebral volume and surface area (Rogers et al., 2010). The positive correlation between GI and cerebral volume and surface observed across species is, therefore, unlikely to come from a common set of genes. Certain human pathologies further demonstrate that genetic mutations affecting gyrencephaly may have limited effect on cerebral volume (e.g., lissencephaly, polymicrogyria) or cerebral volume on gyrencephaly (e.g., microcephaly, megalencephaly). The second stage of gyrification in humans is marked by the prenatal emergence of small sulci and dimples—generated independently of cerebral gyri and accompanied by a major increase in brain weight—and the postnatal growth of sulcal length and depth (Sawada et al., 2012a). Unlike Stage 1, this stage is not correlated with increases in cerebral volume. Rather, patterns in monkeys showing considerable increases in sulcal infolding in the occipital region and secondary and tertiary sulci formation in the frontoparietal region indicate that this period may define species-specific topography of gyri (Fukunishi et al., 2006; Kashima et al., 2008; Sawada et al., 2010, 2012a). For example, increased sulcal infolding in the frontal region of humans (Dubois et al., 2008) compared to macaques (Sawada et al., 2010) underscores the numerous human-specific adaptations to the prefrontal cortex (e.g., Sherwood et al., 2006; Bianchi et al., 2012); and disproportionate inter-indiviual variation in humans in the anterior prefrontal cortex further underscores the phylogenetic recentness and plasticity of this region (Huttner et al., 2005). The terminus of gyrencephaly, too, shows species-specificity: degree of gyrencephaly stabilizes in baboons around birth (Kochunov et al., 2010), while in macaques and humans it reaches a maximum around 1 year after birth (Sawada et al., 2012a). The wide-ranging conservation of gyrencephalic patterning, which cannot be explained simply as a physiological consequence of neocortical expansion, suggests that genetic mechanisms play an important—albeit likely indirect—role in the specification of cortical folding (Rakic, 1988). These genes may either programmatically shape the topology of germinal zones during cortical growth to anticipate gyral and sulcal formation (Smart and McSherry, 1986; Régis et al., 2005) or specify patterns of fiber connectivity to differentially effect tension at the developing cortex (Van Essen, 1997; Hilgetag and Barbas, 2006). The high heritability of early-forming gyri, as well as the species-specific distribution of late-forming sulci, support a scenario in which gyrencephalic tinkering may be accomplished through selection on axonal tension, but that establishment of primary gyri is determined by ventricular (VZ) and subventricular zone (SVZ) organization during cortical development.

## 3. Subventricular expansion and the establishment of gyri

**Figure 2:**
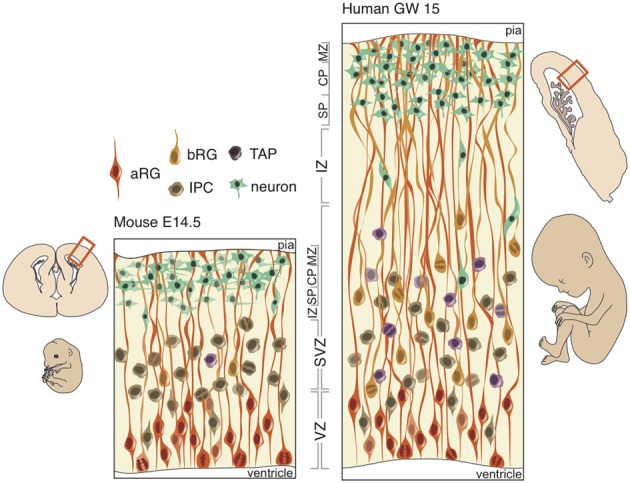
**Schematic of neural progenitors in the developing neocortex in mouse (left) and human (right).** Polarized progenitors (bRG and aRG) are depicted with processes extending to the apical (bottom) and/or basal (top) surface. Non-polarized cells (IPCs and TAPs) divide exclusively in the SVZ in both mouse and human. The human SVZ is relatively expanded compared to the mouse and divided into an outer (OSVZ) and inner (ISVZ) region. CP, cortical plate; IZ, intermediate zone; MZ, marginal zone; SP, subplate.

The emergence of new structures is typically limited to selection on existing developmental pathways. Minor perturbations in timing or cell-type proportions may result in major phenotypic adaptations (e.g., delayed retinal neurogenesis in nocturnal vs. diurnal monkeys or the preponderance of basal or apical neurogenesis in larger- and smaller-brained species). Notwithstanding, there are quite divergent developmental pathways able to generate nearly identical phenotypes (e.g., gastrulation, neural crest formation, and germ cell formation). But in either case, we may assume that selection at the gross morphological level is complemented by adaptations in developmental processes. Therefore, any understanding of the appearance and distribution of cortical folds must be gleaned from a comparison of neural progenitors during development across taxa (Figure 2).

At the onset of neurogenesis, neuroepithelial cells forming a pseudo-stratified epithelium divide rapidly and symmetrically, thus expanding the progenitor pool that will directly or indirectly generate all of the excitatory neurons in the neocortex. As neurogenesis proceeds and the epithelium thickens, neuroepithelial cells, while retaining their apical and basal contacts (Huttner and Brand, 1997; Farkas and Huttner, 2008), begin to express astroglia-specific markers (Campbell and Götz, 2002; Kriegstein and Alvarez-Buylla, 2009), lose their tight junctions and elongate (Kelava and Huttner, 2012). These apical radial glia (aRG) perform interkinetic nuclear migration (Taverna and Huttner, 2010), like neuroepithelial cells, and divide asymmetrically at the apical surface of the VZ (Götz and Huttner, 2005; Fietz and Huttner, 2011; Lui et al., 2011) in order to produce a neuron, intermediate progenitor (IP), or basal radial glia (bRG) (Miyata et al., 2001, 2004; Noctor et al., 2001, 2004; Haubensak et al., 2004; Fietz et al., 2010; Hansen et al., 2010; Reillo et al., 2011). IPs and bRG, like neurons, delaminate from the apical surface and translocate their nucleus to the basal region of the VZ to form the second germinal layer, the SVZ, where non-polar IPs self-consume to produce two neurons and unipolar bRGs generate neurons asymmetrically via IPs or transit-amplifying progenitors (TAPs) (Fietz and Huttner, 2011; Franco and Müller, 2013).

In gyrencephalic species, such as the human and ferret, an abundance of basal-oriented progenitors form not only the SVZ, but subdivide the SVZ into an outer (OSVZ) and inner (ISVZ) region (Smart et al., 2002), each with a distinct expression profile (Fietz et al., 2012). The presence of an OSVZ populated by bRG is thought to be necessary for gyrencephaly: lissencephalic species (e.g., mouse, rat, rabbit) lack this derived region, whereas gyrencephalic species (e.g., human, macaque, ferret) maintain this region. But neither the presence of bRG, which constitute a small minority of SVZ progenitors in the mouse (Shitamukai et al., 2011; Wang et al., 2011), nor an abundance of bRG, which exist in comparable proportions in the lissencephalic marmoset and gyrencephalic human (Kelava et al., 2012), is sufficient for developing a folded neocortex. Several lines of evidence and hypothetical modeling may evince which neurobiological features are both necessary and sufficient for development of a gyrencephalic neocortex.

## 4. Axonal tension and late-stage plasticity in cortical folding

The first cortico-cortical and cortico-subcortical tracts emerge during development of the preplate. As radial pathways across the cortical mantle gradually regress, the subplate forms and thalamo-cortical fibers advance into the cortical plate and cortico-cortical pathways emerge (Kostovic and Rakic, 1990; De Carlos and O'Leary, 1992; Kostović and Jovanov-Milosević, 2006; Kostović and Judas, 2010). In humans, both the radial organization of fiber tracts and establishment of pathways proceed along a posterodorsal→anteroventral gradient, with gyri formation beginning at the parieto-occipital and central sulci during GW24 (Takahashi et al., 2012). One of the earliest suggested and most widely debated hypotheses of a developmental cause for folding focuses on the mechanical tension of axons (Van Essen, 1997). The so-called tension-based hypothesis states that strong, tangentially organized cortico-cortical and weak, radially organized cortico-subcortical pathways, in an effort to minimize the distance between interconnected regions, cause the outward and inward folding of the cortex, respectively.

**Figure 3:**
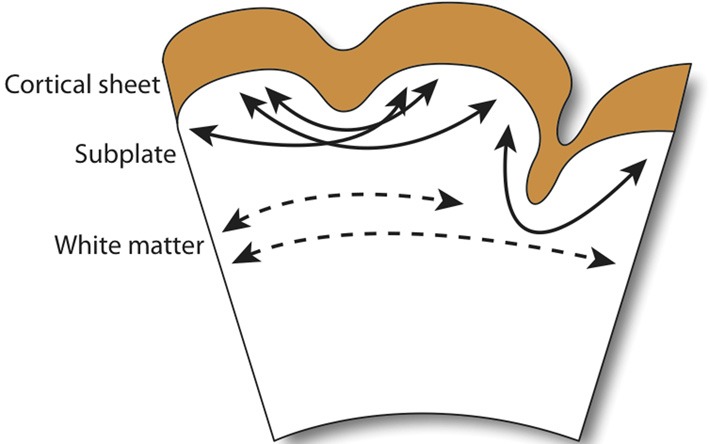
**Observed axonal tension across neocortical gyri.** Axonal tension (arrows) is distributed circumferentially across the subcortical white matter (dashed arrows), but radially in the subplate and gyral folds (filled arrows). Contrary to the connectivty-driven hypothesis (see section 4), circumferential tension is not observed across neocortical gray matter (Xu et al., 2010).

A recent extension of this hypothesis, which ascribes axonal tensions through the white matter the responsibility of pulling inward the cortical surface, proposes that cortical folding is a function of white matter connectivity (Mota and Herculano-Houzel, 2012). While the emergence of primary sulci with long associative fiber tracts is conserved in gyrencephalic species, as is the close correlation between white matter volume and gyrencephaly during development, no direct connection between gyrification and white matter myelination has been observed (Neil et al., 1998). More importantly, the crucial assumption in tension-based hypotheses—that axonal tension is directed across gyri – finds little evidence in its defense (Figure 3). Work in the ferret has shown that, while axons are under considerable tension in the developing brain, the tension is predominantly located in subcortical axon bundles, too deep to affect folding at the surface, and that there is no significant circumferential axonal tension in developing gyri (Xu et al., 2010). In humans, no relationship is observed between gyral formation and the establishment of cortico-cortical fiber pathways (Takahashi et al., 2012). Therefore, axonal tension is unlikely to causally affect cortical folding. However, radial tension within gyri, regulated by white matter connectivity, may limit expansion of the cortex and thereby mediate the shape of the cortical surface (Toro and Burnod, 2005).

Regional variations in axonal tension across the cortex have been suggested to affect cortical shape and influence local folding patterns (Hilgetag and Barbas, 2006; Toro et al., 2008), indicating that axonal tension is either the driving force behind late-stage increases in species-specific gyrification or that early-stage tension forces—too small to drive cortical folding by mechanical deformation—may, nonetheless, provide feedback signals that trigger patterns of differential growth in the germinal zone (see Beloussov, 1998). The coincident emergence of primary sulci with long associative fiber tracts lends support to the latter scenario, wherein the subplate zone plays host to interactions between developing fiber tracts and the production and migration of immature neurons (Kostovic and Rakic, 1990; Armstrong et al., 1995). On the other hand, axonal tension is not observed to induce morphological deformations (Knutsen et al., 2013); so, regional variation in cortical tension, proceeding from a topology of gyri and sulci established by differential gray matter (GM) growth, is more likely to only tinker with late-stage gyrencephaly. Minor intraspecific differences in gyri and sulcal formation, particularly in the late-forming prefrontal cortex, support this scenario (Toro et al., 2008).

## 5. Expansion of the OSVZ increases cortical surface area

**Figure 4:**
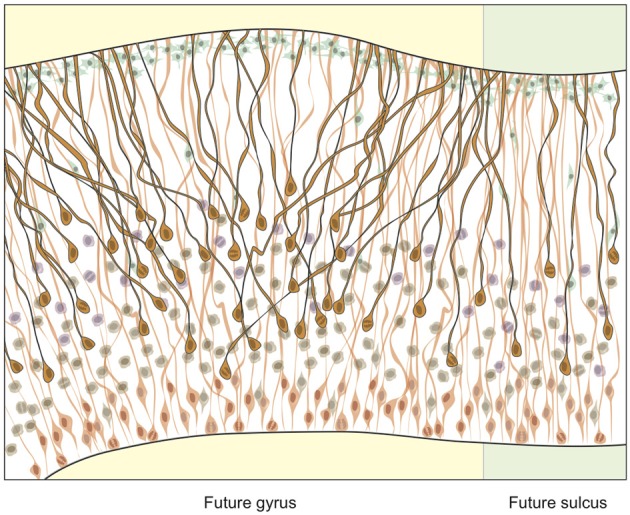
**Basal fibers extending to the cortex during development.** The density of progenitors in the proliferative basal compartment is increased and the angle of migration of their fibers more oblique at sites of developing gyri compared to sulci. In lissencephalic species, the basal compartment is scarcely populated by proliferating progenitors and fibers migrate in parallel to the developing cortex.

The fibers of polarized progenitors provide scaffolding to guide migrating neurons to the developing cortex. In the OSVZ, the scaffolding of bRG resembles a fan, which modifies the trajectory of migrating neurons by driving them to expand conically (Figure 4) (Fietz and Huttner, 2011; Lui et al., 2011; Borrell and Reillo, 2012). This, in turn, increases cortical surface area; and experimentally increasing or decreasing cortical surface area during development leads to the production or reduction of gyri, respectively (Reillo et al., 2011). While the caudal→rostral gradient of cortical folding tends to mirror the transverse gradient of neurogenesis (Smart and McSherry, 1986), no gyrencephalic species has a uniform distribution of gyri and sulci, but a pattern that reflects both functional specialization and phylogenetic inheritance. Therefore, the topology of gyri should be reflected in the distribution and mitotic activity of OSVZ progenitors in the developing neocortex. And so it is. In the cat, the density of OSVZ mitoses is three-fold higher in the prospective parietal compared to temporal cortex, reflecting the higher degree of folding and expanded surface area in the former compared to the latter region; in the ferret, the density of OSVZ mitoses is three-fold higher in the prospective splenial gyrus than lateral sulcus, reflecting the relative conical expansion and cortical folding of those regions; and in the human and monkey, OSVZ mitoses are most abundant in the highly folded parietal and temporal regions (Lukaszewicz et al., 2005; Reillo et al., 2011). The evidence suggests, therefore, that OSVZ progenitors accumulate and/or cycle faster in regions that will undergo the greatest cortical folding.

The degree of cissoidian radial fiber divergence, which drives 3D conical expansion of the cortical surface, increases exponentially during neurogenesis in prospective gyral regions, but remains mostly parallel in smooth regions, as it does in lissencephalic species (Lui et al., 2011; Borrell and Reillo, 2012). Importantly, it is not the production of neurons but the divergence of radial fibers (which may be an evolutionary response to increases in neuron production) that drives conical expansion. Enucleation studies in the ferret show how a reduction in the proliferation of bRG leads to a smaller, but no less gyrified, splenial gyrus (Reillo et al., 2011). The mechanistic, and likely genetic (see section 2), dissociation of neuron production and cortical folding is also clear in disease phenotypes. Pachygyria, for example, is characterized by a decrease in cortical surface area, but not neuron number (Ross and Walsh, 2001), whereas the decrease in neuron number in microcephaly is not accompanied by a commensurate loss in cortical folding (Bond et al., 2002). However, the dissociation of neuron production and cortical folding in development does not necessarily imply that these traits were subject to distinct selection pressures. On the contrary, the ubiquity of enlarged, gyrencephalic brains across the mammalian phylogeny, and the near absence of large-brained lissencephalic species, strongly suggests that neocortical expansion and folding evolved in concert.

## 6. Gyrencephaly and cortical thinning as mechanistic responses to evolutionary increases in neuron production

**Figure 5:**
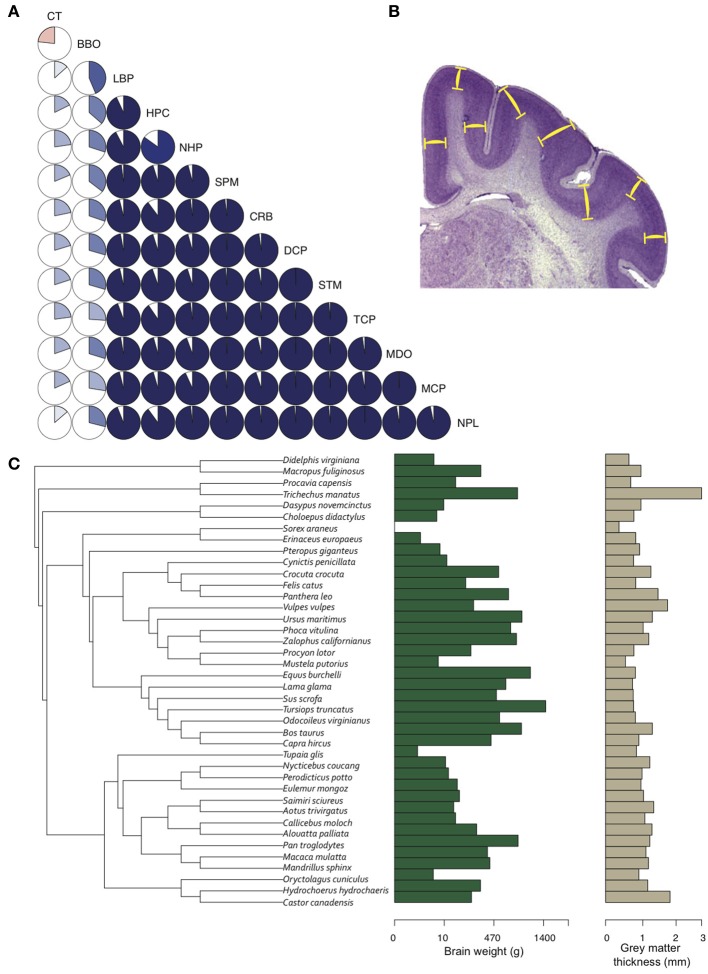
**Gray matter cortical thickness varies with brain regions and phylogeny. (A)** Twelve brain region volumes and GM thickness presented in a pie-chart matrix of positive (blue gradient) and negative (red gradient) correlations. Note that all brain region volumes - except BBO, which is a developmentally and functionally separate region - show very high (*R*^2^ > 0.8) positive correlations, whereas cortical thickness is lowly (*R*^2^ < 0.4) correlated with all brain region volumes. BBO, olfactory bulb; CRB, cerebellum; CT, cortical thickness; DCP, diencephalon; HPC, hippocampus; LBP, piriform lobe; MCP, mesencephalon; MDO, medulla oblongata; NHP, neurohypophysis; NPL, neopallial; SPM, septum; STM, striatum; TCP, telencephalon. Volumetric data from Stephan et al. (1981). **(B)** GM thickness is measured as the average distance between layers I and VI (yellow bars) in a systematic random sample of the neocortex. **(C)** A phylogenetic tree of 40 mammal species (Bininda-Emonds et al. 2007) showing the distribution of brain weight (log10 + 1) and GM thickness (log10 + 1) across species. GM thickness in all species was measured with Fiji (Schindelin et al., 2012) on slides from brainmuseum.org. See Lewitus et al. (2013) and Table A1 for neuroanatomical data in **(C)**.

Given two brains of equal radial dimensions, the more folded specimen tends to have a thinner cortex (Hofman, 1985; Pillay and Manger, 2007). In humans, a thin and extensively folded neocortex is characteristic of polymicrogyria (Rakic, 1988; Chang et al., 2004) and may manifest in schizophrenia (Harrison, 1999; Palaniyappan and Liddle, 2012), Williams syndrome (Gaser et al., 2006), and autism (Jou et al., 2010). Across species, the most gyrencephalic taxa (cetartiodactyla) also have the thinnest cortices (Manger et al., 2012). Nonetheless, the relationships between brain volume, gyrencephaly, and GM cortical thickness, in development and evolution, remain elusive (Figure 5).

**Figure 6:**
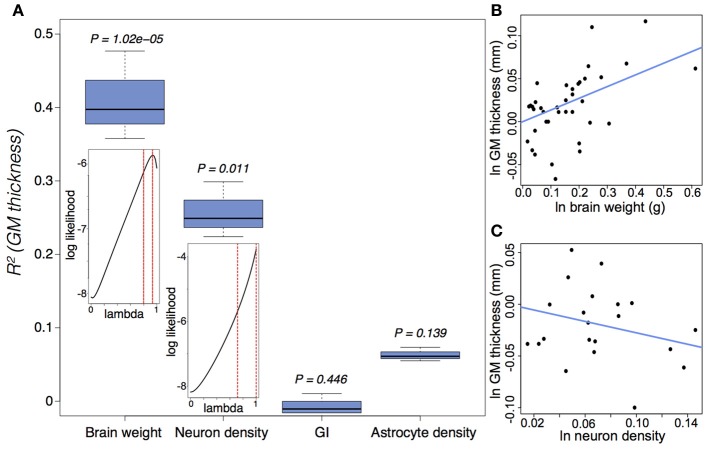
**Gray matter (GM) thickness is a function of brain weight and neuron density. (A)** Variation in GM thickness can be significantly explained by brain weight [*F*_(2, 37)_ = 22.58, *P* = 3.9 × 10^−7^] and neuron density [*F*_(2, 20)_ = 7.96, *P* = 0.003], but not by either GI [*F*_(2, 38)_ = 0.066, *P* = 0.936] or astrocyte density [*F*_(2, 20)_ = 2.37, *P* = 0.119]. The insets suggest a strong phylogenetic signal (Pagel, 1999), tantamount to a random walk, in the scaling of GM thickness as a function of brain weight (lambda = 0.89^(+0.07)^_(−0.09)_) and neuron density (lambda = 0.88^(+0.12)^_(−0.17)_). **(B,C)** Ln-transformed phylogenetically independent contrasts with regression through the origin for GM thickness as a function of **(B)** brain weight and **(C)** neuron density. GM thickness scales positively as a function of brain weight (e^0.136 ± 0.027^) and negatively as a function of neuron density (e^−0.276 ± 0.098^). Cell densities pertain to gray matter counts in the visual cortex from Lewitus et al. (2012). See (Lewitus et al., 2013) and Table A1 for neuroanatomical data.

GM thickness and GI—like brain volume, cortical surface area, and gray matter volume—are heritable traits (Panizzon et al., 2009; Eyler et al., 2012). But while brain volume, cortical surface area, and gray matter volume show high levels of statistical and genetic correlation within a population, GM thickness and GI are lowly or negatively correlated with most neuroanatomical traits (Rogers et al., 2010; Winkler et al., 2010). In mammalian evolution, we also find a somewhat chaotic correlative pattern of GM thickness (Figure 5). Cetaceans are the most gyrencephalic mammals and exhibit a thin cortex (<1.75 mm) and low neuron density (<65,000 per mm^3^) (Hof et al., 2005; Kern et al., 2011); but despite a magnitude of variation in brain volume across cetacean species, GI values remain nearly constant. Pinnipeds, the aquatic carnivores, likewise show very high levels of gyrencephaly (Manger et al., 2012). So perhaps adaptation to an aquatic environment releases a constraint on evolving increasingly folded cortices (see Butti et al., 2011). However, the manatee, the only other aquatic mammal, has a relatively large, lissencephalic brain and a thick cortex (~3 mm) (Reep et al., 1989; Reep and O'Shea, 1990; Marshall and Reep, 1995). Among terrestrial mammals, artiodactyls have the highest GI values, as well as distinctly thin cortices and low neuron densities compared to primates and carnivores (Chow, 1950), whereas the relatively large-brained beaver, like the manatee, is lissencephalic (Pillay and Manger, 2007). A loose negative correlation between GI and relative (i.e., corrected for neocortical volume) ventricular volume [*F*_(1, 30)_ = 3.834, *P* = 0.06] may explain the large ventricles and smooth cortices of the beaver and manatee. Furthermore, our analyses find significant scaling relationships between GM thickness and both brain weight and neuron density (Figure 6) (Harrison et al., 2002). These data support the observed convergence of GM thinning in large-brained species, but not the lack of correlation between GM thickness and other neuroanatomical variables within human and other primate populations (see above). If the genes, and therefore selection pressures, mediating GM thickness and folding are independent of those mediating other brain variables, as our and previous analyses suggest, then we should consider a developmental scenario wherein cortical folding and thinning become advantageous to selection for increases in neuron number.

There is a 1000-fold difference in cortical neuron number between mouse and human, but only a 10-fold difference in the length of the neurogenic period. The increase in neuron number in human, therefore, means an exponential amplification of neuron generation. As discussed in section 3, neurons in the human and other large-brained species are generated primarily in the OSVZ, where immature neurons migrate to the cortical plate along fibers provided by bRG. It is the divergence of these fibers that drives conical expansion and ultimately gyrification of the neocortex (see section 5). However, the divergence of radial fibers exiting the OSVZ only organize the migration of neurons to the cortex, allowing them to fan out across an expanding surface rather than continue to populate an overcrowding cortical column (i.e., radial fiber divergence has adapted to accommodate selection for increased neuron generation). The ubiquity of gyrencephaly across mammalian orders, absent any genetic correlation between brain volume and GI (see above), suggests that the mechanistic ability for radial fibers in the OSVZ to diverge in response to rapid increases in neuron generation is either extremely adaptable or deeply homologous (i.e., the conical expansion of fibers is likely constrained by mechanistic limitations or by a conserved developmental toolbox that makes any other solution to the problem of increasing neuron generation deleteriously demanding). But in either case, cortical folding is simply a conserved, mechanistic response to selection for an increased generation of neurons per neurogenic period. In the next section, we will discuss how maintenance of a proliferative niche in the OSVZ may underpin such increases in neuron generation.

## 7. Maintenance of a basal proliferative niche during peak neurogenesis

Conical expansion of the SVZ into outer and inner regions is a hallmark of increased neurogenic proliferative capacity (Smart et al., 2002). It is likely necessary—but not sufficient (Kelava et al., 2012)—to generate a gyrencephalic neocortex. In the human OSVZ, bRG cells may generate neurons via TAPs, progenitor cells capable of multiple rounds of proliferation (Hansen et al., 2010); and while TAPs are putatively present in other large-brained, highly gyrencephalic species, they have not been observed in significant numbers or with comparable proliferative capacity in lissencephalic species (Wang et al., 2011). Intrinsic factors, such as the expression level and inheritance of certain transcription factors (e.g., Pax6, Sox2) likely play a role in the proliferative capacity of bRG and TAPs, but there is accumulating evidence that extrinsic factors distinguish the behavior of progenitors in the basal compartment between lissencephalic and gyrencephalic species.

Extracellular matrix has been implicated in expansion of the SVZ (Barros et al., 2011; Fietz et al., 2012). For example, interference with integrin signaling, a major part of ECM-derived signaling, results in a reduced number of bRG without affecting the TAP/IP population (Fietz et al., 2010). This suggests that the proliferative capacity of bRGs, compared to TAPs/IPs, depends on integrin signaling maintained via the basal process. Furthermore, there is a denser invasion of incoming thalamic fibers in the SVZ of gyrencephalic compared to lissencephalic species. These fibers secrete proliferation-promoting factors (Kriegstein and Alvarez-Buylla, 2009; Dehay et al., 2001) and subdivide the SVZ into an outer and inner region in gyrencephalic species (Smart et al., 2002). Work in the mouse has shown that blood vessels in the SVZ, which have basal lamina, establish a proliferative niche in their vicinity (Javaherian and Kriegstein, 2009; Stubbs et al., 2009), so vascularization of the developing neocortex is also likely to be integral to the establishment and maintenance of a proliferative SVZ. While it remains unknown which factors are secreted by blood vessels, basal processes, and other ECM vehicles to determine the proliferative capacity of the basal compartment, transcriptome analyses of the developing neocortex in human and mouse have revealed an enrichment of ECM-related transcripts, not only in the OSVZ compared to the VZ, but also in the human OSVZ compared to the mouse SVZ (Arai et al., 2011; Fietz et al., 2012), providing clear evidence for an interplay between ECM signaling, an expanded basal compartment, and a large, gyrencephalic neocortex.

## 8. Conclusion

Brain size is subject to significant heritability. As such, selection pressures directing brain evolution in humans have ranged from tool-making abilities to diet to long-distance running [reviewed in Healy and Rowe (2007)]. While none of these pressures is likely to be solely responsible for human neocortical expansion—nor can any of them be incorporated into a general theory of mammalian neocortical expansion—the fact remains: the neocortex has expanded many times in mammalian evolution; and the features underwriting that expansion may ultimately be traced back to neurogenic changes at the cellular level. What remains to be understood, however, is which features are highly constrained and which features have been repeatedly implicated in neocortical evolution.

Adult mammalian brains are not identical at the cellular level. Phylogenetic differences in the density of cortical columns and in the morphology and biochemistry of neurons have been identified in most orders (e.g., Beaulieu, 1993; Peters and Yilmaz, 1993; Nimchinsky et al., 1999; Preuss and Coleman, 2002; Hof and Van der Gucht, 2007; Herculano-Houzel, 2011). The configuration of structural and functional topographical maps that constitute the mammalian brain, too, has seen many evolutionary examples of proliferation, addition, and segregation [reviewed in Krubitzer and Seelke (2012)]. Therefore, universal modular architecture does not exist for the mammalian neocortex and neocortical size may not fairly be considered as an index of general functional capacity. Differential growth across the neocortex and between species, however, may tell us how variation in neocortical size is achieved, even if it will not necessarily inform us of the environmental selection pressures effecting that variation. Here, we have taken a reductionist approach by claiming that a gross neuroanatomical feature (neocortical folding) may signify differences in neurogenic programming both within an individual and across species. We have made this claim based on evidence that neocortical size is determined before any neuronal connections are established and on the assumption that the formation of neocortical gyri is the result of an interaction between selection pressures in cognitive or sensory behavior and the cell-biological properties of neural progenitors throughout neurogenesis. Neocortical size is determined by neurogenic programming (i.e., the distribution of progenitor-type populations and the differential regulation of those populations during neurogenesis). Some neocortical regions may have higher neuron numbers or densities requiring greater degrees of local modulation and control (Collins et al., 2010; Collins, 2011; Bianchi et al., 2012). In these regions, tremendous perinatal increases in astrocytes and oligodendrocytes will drive the morphological expansion of neocortical regions. Specializations in behavior are known to be complemented by cellular or molecular enhancements in the regions of the brain mediating those specializations (Krubitzer, 2007). The enlargement of Meynert cells in the visual cortex of monkeys compared to carnivores, for example, is thought to represent the evolution of a cellular substrate for specialized sensorimotor capacities related to eye-hand movements that are highly developed in monkeys compared to carnivores (le Gros Clark, 1942; Sherwood et al., 2003). Similarly, the introduction of acoustic noise to rat pups has been shown to alter the cortical magnification of particular neuronal frequencies in the primary auditory cortex (Chang and Merzenich, 2003), showing that even within an individual behavioral and cellular adaptations are tightly linked. In the case of mammalian neocortical expansion, we observe increased vascularization of the neocortical germinal zone, subdivision of the SVZ into an outer and inner region, expansion of the OSVZ, upregulation of ECM signaling to abventricular progenitors, and increased proliferative capacities of non-polar progenitors in the basal compartment of large-brained, gyrencephalic species. We think that these features are correlated in both development and evolution and that any variation between individuals or species in neocortical morphology will not only be underwritten by changes in neurogenic programming but will also be constrained by limitations imposed by the mammalian neurogenic program.

### Conflict of interest statement

The authors declare that the research was conducted in the absence of any commercial or financial relationships that could be construed as a potential conflict of interest.

## Appendix

**Table A1:** **Neuroanatomical parameters in 40 mammal species**^*****^.

**Species**	**Brain weight (g)**^**a**^	**Neuron density (per mm**^**3**^**)**^**b**^	**Astrocyte density (per mm**^**3**^**)**^**b**^	**Gray matter thickness (mm)**^**c**^	**Ventricle (1 and 2) volume (mm**^**3**^**)**^**a**^	**GI**^**d**^
*Castor canadensis*	41.17	NA	NA	1.82	NA	1.02
*Hydrochoerus h.*	63.5	NA	NA	1.19	NA	1.3
*Oryctolagus cuniculus*	6.5	NA	NA	0.94	NA	1.15
*Erythrocebus patas*	105.65	416869	154882	NA	561	1.91
*Miopithecus talapoin*	39.1	NA	NA	NA	262	1.74
*Mandrillus sphinx*	NA	263027	138038	1.21	NA	2.14
*Lophocebus albigena*	103.38	NA	NA	NA	742	1.87
*Macaca mulatta*	89.22	422149	113783	1.14	834	1.79
*Piliocolobus badius*	76.75	NA	NA	NA	455	1.81
*Pygathrix nemaeus*	84.83	NA	NA	NA	911	1.64
*Gorilla gorilla*	477.44	144544	138038	NA	3608	2.26
*Pan troglodytes*	392.06	208930	123027	1.25	1899	2.46
*Hylobates lar*	101.52	NA	NA	NA	555	1.86
*Alouatta palliata*	52.75	176349	49168	1.31	NA	1.33
*Lagothrix lagotricha*	95.58	NA	NA	NA	1090	1.97
*Callicebus moloch*	19	467735	125893	1.11	NA	1.25
*Aotus trivirgatus*	17.4	410950	59930	1.36	105	1.31
*Callimico goeldii*	10.95	NA	NA	NA	48	1.26
*Callithrix jacchus*	7.61	NA	NA	NA	52	1.17
*Saguinus midas*	10.5	NA	NA	NA	251	1.2
*Saimiri sciureus*	22.98	478630	117490	1.07	299	1.46
*Microcebus murinus*	1.85	190546	112202	NA	11	1.1
*Cheirogaleus major*	6.43	NA	NA	NA	83	1.15
*Cheirogaleus medius*	3.01	186209	109648	NA	25	1.11
*Avahi laniger*	10.65	NA	NA	NA	172	1.26
*Avahi occidentalis*	9.69	NA	NA	NA	74	1.15
*Propithecus verreauxi*	26.9	NA	NA	NA	231	1.35
*Indri indri*	37.35	NA	NA	NA	330	1.46
*Daubentonia m.*	44.89	NA	NA	NA	392	1.25
*Eulemur fulvus*	28.1	NA	NA	NA	194	1.46
*Eulemur mongoz*	20.75	234423	138038	1	NA	1.33
*Varecia variegata*	49.83	NA	NA	NA	299	1.32
*Lepilemur ruficaudatus*	7.5	NA	NA	NA	77	1.14
*Perodicticus potto*	13.54	NA	NA	1.03	127	1.27
*Loris tardigradus*	6.63	NA	NA	NA	52	1.29
*Nycticebus coucang*	11.73	109648	53703	1.25	142	1.21
*Galago senegalensis*	4.8	338844	151356	NA	40	1.17
*Otolemur crassicaudatus*	10.6	NA	NA	NA	147	1.26
*Galago demidoff*	3.35	NA	NA	NA	30	1.21
*Tupaia glis*	3.03	131826	107152	0.87	NA	1.06
*Capra hircus*	106	NA	NA	0.94	NA	2.28
*Bos taurus*	462	NA	NA	1.32	NA	2.53
*Odocoileus virginianus*	160	NA	NA	0.84	NA	2.27
*Tursiops truncatus*	1489	147911	229087	0.79	NA	4.76
*Sus scrofa*	137.65	48978	70795	0.78	NA	2.16
*Lama glama*	216.77	NA	NA	0.76	NA	2.7
*Equus caballus*	712	NA	NA	0.84	NA	2.8
*Mustela putorius*	8.25	NA	NA	0.56	NA	1.75
*Procyon lotor*	40.02	104713	83176	0.8	NA	1.85
*Zalophus californianus*	363	30903	57544	1.22	NA	2.52
*Phoca vitulina*	273.75	NA	NA	1.06	NA	2.38
*Ursus maritimus*	472.68	44668	95499	1.32	NA	2.04
*Vulpes vulpes*	45.63	81283	77625	1.75	NA	1.8
*Panthera leo*	247.21	NA	NA	1.48	NA	1.85
*Felis catus*	31.18	114815	22909	0.85	NA	1.5
*Crocuta crocuta*	153.27	63096	79433	1.28	NA	1.74
*Cynictis penicillata*	12.51	141254	123027	0.79	NA	1.35
*Pteropus giganteus*	9	NA	NA	0.96	NA	1.25
*Erinaceus europaeus*	3.5	194984	128825	0.85	NA	1
*Sorex araneus*	0.2	338844	295121	0.38	NA	1
*Choloepus didactylus*	7.7	NA	NA	0.8	NA	1.38
*Dasypus novemcinctus*	10.75	NA	NA	1	NA	1.07
*Trichechus manatus*	382	51286	97724	2.71	NA	1.02
*Procavia capensis*	19.17	NA	NA	0.71	NA	1.37
*Macropus fuliginosus*	64.8	NA	NA	1	NA	1.41
*Didelphis virginiana*	6.72	NA	NA	0.66	NA	1.12

* All data for adult.

a Stephan et al., 1981.

b Lewitus et al., 2012.

c See Figure 5.

d Lewitus et al., 2013.

## References

[B1] Arai Y. Pulvers J. N. Haffner C. Schilling B. Nüsslein I. Calegari F. (2011). Neural stem and progenitor cells shorten s-phase on commitment to neuron production. Nat. Comm. 2:154 10.1038/ncomms1155 21224845 PMC3105305

[B2] Armstrong E. Schleicher A. Omran H. Curtis M. Zilles K. (1995). The ontogeny of human gyrification. Cereb. Cortex, 5, 56–63 10.1093/cercor/5.1.56 7719130

[B3] Barnette A. R. Neil J. J. Kroenke C. D. Griffith J. L. Epstein A. A. Bayly P. V. (2009). Characterization of brain development in the ferret via MRI. Pediatr. Res. 66, 80–84 10.1203/PDR.0b013e3181a291d9 19287340 PMC3384539

[B4] Barros C. S. Franco S. J. Müller U. (2011). Extracellular matrix: functions in the nervous system. Cold Spring Harb. Perspect. Biol. 3:a005108 10.1101/cshperspect.a005108 21123393 PMC3003458

[B5] Bayer S. A. Altman J. (1991). Neocortical Development. New York, NY: Raven Press

[B6] Beaulieu C. (1993). Numerical data on neocortical neurons in adult rat, with special reference to the GABA population. Brain Res. 609, 284–292 10.1016/0006-8993(93)90884-P 8508310

[B7] Beloussov L. V. (1998). The Dynamic Architecture of a Developing Organism: an Interdisciplinary Approach to the Development of Organisms. Dordrecht: Kluwer, 145–163

[B8] Bianchi S. Stimpson C. D. Bauernfeind A. L. Schapiro S. J. Baze W. B. McArthur M. J. (2012). Dendritic morphology of pyramidal neurons in the chimpanzee neocortex: regional specializations and comparison to humans. Cereb. Cortex. [Epub ahead of print]. 10.1093/cercor/bhs239 22875862 PMC3767963

[B9] Bond J. Roberts E. Mochida G. H. Hampshire D. J. Scott S. Askham J. M. (2002). ASPM is a major determinant of cerebral cortical size. Nat. Genet. 32, 316–320 10.1038/ng995 12355089

[B10] Borrell V. Reillo I. (2012). Emerging roles of neural stem cells in cerebral cortex development and evolution. Dev. Neurobiol. 72, 955–971 10.1002/dneu.22013 22684946

[B11] Butti C. Raghanti M. A. Sherwood C. C. Hof P. R. (2011). The neocortex of cetaceans: cytoarchitecture and comparison with other aquatic and terrestrial species. Ann. N.Y. Acad. Sci. 1225, 47–58 10.1111/j.1749-6632.2011.05980.x 21534992

[B12] Campbell K. Götz M. (2002). Radial glia: multi-purpose cells for vertebrate brain development. Trends Neurosci. 25, 235–238 10.1016/S0166-2236(02)02156-2 11972958

[B13] Chang B. S. Piao X. Giannini C. Cascino G. D. Scheffer I. Woods C. G. (2004). Bilateral generalized polymicrogyria (BGP): a distinct syndrome of cortical malformation. Neurology 62, 1722–1728 10.1212/01.WNL.0000125187.52952.E9 15159468

[B14] Chang E. F. Merzenich M. M. (2003). Environmental noise retards auditory cortical development. Science 300, 498–502 10.1126/science.1082163 12702879

[B15] Chi J. G. Dooling E. C. Gilles F. H. (1977). Gyral development of the human brain. Ann. Neurol. 1, 86–93 10.1002/ana.410010109 560818

[B16] Chow K. (1950). Cell ratios in the thalamocortical visual system of macaca mulatta. J. Comp. Neurol. 92, 227–239 10.1002/cne.900920208 15415473

[B17] Clowry G. Molnár Z. Rakic P. (2010). Renewed focus on the developing human neocortex. J. Anat. 217, 276–288 20979582 10.1111/j.1469-7580.2010.01281.x PMC2992407

[B18] Collins C. E. (2011). Variability in neuron densities across the cortical sheet in primates. Brain Behav. Evol. 78, 37–50 10.1159/000327319 21691046

[B19] Collins C. E. Airey D. C. Young N. A. Leitch D. B. Kaas J. H. (2010). Neuron densities vary across and within cortical areas in primates. Proc. Natl. Acad. Sci. U.S.A. 107, 15927–15932 10.1073/pnas.1010356107 20798050 PMC2936588

[B20] De Carlos J. A. O'Leary D. D. (1992). Growth and targeting of subplate axons and establishment of major cortical pathways. J. Neurosci. 12, 1194–1211 1556593 10.1523/JNEUROSCI.12-04-01194.1992 PMC6575791

[B21] Dehay C. Giroud P. Berland M. Smart I. Kennedy H. (1993). Modulation of the cell cycle contributes to the parcellation of the primate visual cortex. Nature 366, 464–466 10.1038/366464a0 8247154

[B22] Dehay C. Savatier P. Cortay V. Kennedy H. (2001). Cell-cycle kinetics of neocortical precursors are influenced by embryonic thalamic axons. J. Neurosci. 21, 201–214 11150337 10.1523/JNEUROSCI.21-01-00201.2001 PMC6762433

[B23] Dubois J. Benders M. Cachia A. Lazeyras F. Ha-Vinh Leuchter R. Sizonenko S. V. (2008). Mapping the early cortical folding process in the preterm newborn brain. Cereb. Cortex 18, 1444–1454 10.1093/cercor/bhm180 17934189

[B24] Eyler L. T. Chen C.-H. Panizzon M. S. Fennema-Notestine C. Neale M. C. Jak A. (2012). A comparison of heritability maps of cortical surface area and thickness and the influence of adjustment for whole brain measures: a magnetic resonance imaging twin study. Twin. Res. Hum. Genet. 15, 304–314 10.1017/thg.2012.3 22856366 PMC3549553

[B25] Farkas L. M. Huttner W. B. (2008). The cell biology of neural stem and progenitor cells and its significance for their proliferation versus differentiation during mammalian brain development. Curr. Opin. Cell Biol. 20, 707–715 10.1016/j.ceb.2008.09.008 18930817

[B26] Fietz S. A. Huttner W. B. (2011). Cortical progenitor expansion, self-renewal and neurogenesis-a polarized perspective. Curr. Opin. Neurobiol. 21, 23–35 10.1016/j.conb.2010.10.002 21036598

[B27] Fietz S. A. Kelava I. Vogt J. Wilsch-Bräuninger M. Stenzel D. Fish J. L. (2010). OSVZ progenitors of human and ferret neocortex are epithelial-like and expand by integrin signaling. Nat. Neurosci. 13, 690–699 10.1038/nn.2553 20436478

[B28] Fietz S. A. Lachmann R. Brandl H. Kircher M. Samusik N. Schröder R. (2012). Transcriptomes of germinal zones of human and mouse fetal neocortex suggest a role of extracellular matrix in progenitor self-renewal. Proc. Natl. Acad. Sci. U.S.A. 109, 11836–11841 10.1073/pnas.1209647109 22753484 PMC3406833

[B29] Franco S. J. Müller U. (2013). Shaping our minds: stem and progenitor cell diversity in the mammalian neocortex. Neuron 77, 19–34 10.1016/j.neuron.2012.12.022 23312513 PMC3557841

[B30] Fukunishi K. Sawada K. Kashima M. Sakata-Haga H. Fukuzaki K. Fukui Y. (2006). Development of cerebral sulci and gyri in fetuses of cynomolgus monkeys (macaca fascicularis). Anat. Embryol. 211, 757–764 10.1007/s00429-006-0136-7 17072644

[B31] Gaser C. Luders E. Thompson P. M. Lee A. D. Dutton R. A. Geaga J. A. (2006). Increased local gyrification mapped in williams syndrome. Neuroimage 33, 46–54 10.1016/j.neuroimage.2006.06.018 16901723

[B32] Götz M. Huttner W. B. (2005). The cell biology of neurogenesis. Nat. Rev. Mol. Cell Biol. 6, 777–788 10.1038/nrm1739 16314867

[B33] Hansen D. V. Lui J. H. Parker P. R. L. Kriegstein A. R. (2010). Neurogenic radial glia in the outer subventricular zone of human neocortex. Nature 464, 554–561 10.1038/nature08845 20154730

[B34] Harrison K. H. Hof P. R. Wang S. S. (2002). Scaling laws in the mammalian neocortex: does form provide clues to function? J. Neurocytol. 31, 289–298 10.1023/A:1024178127195 12815248

[B35] Harrison P. J. (1999). The neuropathology of schizophrenia. a critical review of the data and their interpretation. Brain 122(Pt 4), 593–624 10.1093/brain/122.4.593 10219775

[B36] Haubensak W. Attardo A. Denk W. Huttner W. B. (2004). Neurons arise in the basal neuroepithelium of the early mammalian telencephalon: a major site of neurogenesis. Proc. Natl. Acad. Sci. U.S.A. 101, 3196–3201 10.1073/pnas.0308600100 14963232 PMC365766

[B37] Healy S. D. Rowe C. (2007). A critique of comparative studies of brain size. Proc. Biol. Sci. 274, 453–464 10.1098/rspb.2006.3748 17476764 PMC1766390

[B38] Herculano-Houzel S. (2011). Not all brains are made the same: new views on brain scaling in evolution. Brain Behav. Evol. 78, 22–36 10.1159/000327318 21691045

[B39] Hilgetag C. C. Barbas H. (2006). Role of mechanical factors in the morphology of the primate cerebral cortex. PLoS Comput. Biol. 2:e22 10.1371/journal.pcbi.0020022 16557292 PMC1409812

[B40] Hof P. R. Chanis R. Marino L. (2005). Cortical complexity in cetacean brains. Anat. Rec. 287A, 1142–1152 10.1002/ar.a.20258 16200644

[B41] Hof P. R. Van der Gucht E. (2007). Structure of the cerebral cortex of the humpback whale, megaptera novaeangliae (cetacea, mysticeti, balaenopteridae). Anat. Rec. (Hoboken) 290, 1–31 10.1002/ar.20407 17441195

[B42] Hofman M. A. (1985). Size and shape of the cerebral cortex in mammals. i. the cortical surface. Brain Behav. Evol. 27, 28–40 10.1159/000118718 3836731

[B43] Huttner H. B. Lohmann G. von Cramon D. Y. (2005). Magnetic resonance imaging of the human frontal cortex reveals differential anterior-posterior variability of sulcal basins. Neuroimage 25, 646–651 10.1016/j.neuroimage.2004.12.008 15784444

[B44] Huttner W. B. Brand M. (1997). Asymmetric division and polarity of neuroepithelial cells. Curr. Opin. Neurobiol. 7, 29–39 10.1016/S0959-4388(97)80117-1 9039800

[B45] Javaherian A. Kriegstein A. (2009). A stem cell niche for intermediate progenitor cells of the embryonic cortex. Cereb. Cortex 19 Suppl. 1, i70–i77 10.1093/cercor/bhp029 19346271 PMC2693531

[B46] Jou R. J. Minshew N. J. Keshavan M. S. Hardan A. Y. (2010). Cortical gyrification in autistic and asperger disorders: a preliminary magnetic resonance imaging study. J. Child Neurol. 25, 1462–1467 10.1177/0883073810368311 20413799 PMC3115701

[B47] Kashima M. Sawada K. Fukunishi K. Sakata-Haga H. Tokado H. Fukui Y. (2008). Development of cerebral sulci and gyri in fetuses of cynomolgus monkeys (macaca fascicularis). II. gross observation of the medial surface. Brain Struct. Funct. 212, 513–520 10.1007/s00429-008-0171-7 18236075

[B48] Kelava I. Huttner W. B. (2012). Neurogenesis in the developing mammalian neocortex, in eLS. John Wiley & Sons, Ltd 10.1002/9780470015902.a0022541

[B49] Kelava I. Reillo I. Murayama A. Y. Kalinka A. T. Stenzel D. Tomancak P. (2012). Abundant occurrence of basal radial glia in the subventricular zone of embryonic neocortex of a lissencephalic primate, the common marmoset callithrix jacchus. Cereb. Cortex 22, 469–481 10.1093/cercor/bhr301 22114084 PMC3256412

[B50] Kern A. Siebert U. Cozzi B. Hof P. R. Oelschläger H. H. A. (2011). Stereology of the neocortex in odontocetes: qualitative, quantitative, and functional implications. Brain Behav. Evol. 77, 79–90 10.1159/000323674 21358169

[B51] Knutsen A. K. Kroenke C. D. Chang Y. V. Taber L. A. Bayly P. V. (2013). Spatial and temporal variations of cortical growth during gyrogenesis in the developing ferret brain. Cereb. Cortex 23, 488–498 10.1093/cercor/bhs042 22368085 PMC3539456

[B52] Kochunov P. Castro C. Davis D. Dudley D. Brewer J. Zhang Y. (2010). Mapping primary gyrogenesis during fetal development in primate brains: high-resolution *in utero* structural MRI study of fetal brain development in pregnant baboons. Front. Neurosci. 4:20 10.3389/fnins.2010.00020 20631812 PMC2896074

[B53] Kostović I. Jovanov-Milosević N. (2006). The development of cerebral connections during the first 20-45 weeks' gestation. Semin. Fetal Neonatal. Med. 11, 415–422 16962836 10.1016/j.siny.2006.07.001

[B54] Kostović I. Judas M. (2010). The development of the subplate and thalamocortical connections in the human foetal brain. Acta Paediatr. 99, 1119–1127 20367617 10.1111/j.1651-2227.2010.01811.x

[B55] Kostovic I. Rakic P. (1990). Developmental history of the transient subplate zone in the visual and somatosensory cortex of the macaque monkey and human brain. J. Comp. Neurol. 297, 441–470 10.1002/cne.902970309 2398142

[B56] Kriegstein A. Alvarez-Buylla A. (2009). The glial nature of embryonic and adult neural stem cells. Annu. Rev. Neurosci. 32, 149–184 10.1146/annurev.neuro.051508.135600 19555289 PMC3086722

[B57] Krubitzer L. (2007). The magnificent compromise: cortical field evolution in mammals. Neuron 56, 201–208 10.1016/j.neuron.2007.10.002 17964240

[B58] Krubitzer L. A. Seelke A. M. H. (2012). Cortical evolution in mammals: the bane and beauty of phenotypic variability. Proc. Natl. Acad. Sci. U.S.A. 109 Suppl. 1, 10647–10654 10.1073/pnas.1201891109 22723368 PMC3386882

[B59] le Gros Clark W. E. (1942). The cells of meynert in the visual cortex of the monkey. J. Anat. 76(Pt 4), 369–376.1 17104906 PMC1252676

[B60] Lewitus E. Hof P. R. Sherwood C. C. (2012). Phylogenetic comparison of neuron and glia densities in the primary visual cortex and hippocampus of carnivores and primates. Evolution 66, 2551–2563 10.1111/j.1558-5646.2012.01601.x 22834752

[B61] Lewitus E. Kelava I. Kalinka A. T. Tomancak P. Huttner W. B. (2013). An adaptive threshold in mammalian neocortical evolution. arXiv 1304.5412

[B62] Lui J. H. Hansen D. V. Kriegstein A. R. (2011). Development and evolution of the human neocortex. Cell 146, 18–36 21729779 10.1016/j.cell.2011.06.030 PMC3610574

[B63] Lukaszewicz A. Savatier P. Cortay V. Giroud P. Huissoud C. Berland M. (2005). G1 phase regulation, area-specific cell cycle control, and cytoarchitectonics in the primate cortex. Neuron 47, 353–364 10.1016/j.neuron.2005.06.032 16055060 PMC1890568

[B64] Manger P. R. Prowse M. Haagensen M. Hemingway J. (2012). Quantitative analysis of neocortical gyrencephaly in african elephants (*Loxodonta africana*) and six species of cetaceans: comparison with other mammals. J. Comp. Neurol. 520, 2430–2439 10.1002/cne.23046 22237903

[B65] Marshall C. D. Reep R. L. (1995). Manatee cerebral cortex: cytoarchitecture of the caudal region in trichechus manatus latirostris. Brain Behav. Evol. 45, 1–18 10.1159/000113381 7866767

[B66] Mayhew T. M. Mwamengele G. L. Dantzer V. Williams S. (1996). The gyrification of mammalian cerebral cortex: quantitative evidence of anisomorphic surface expansion during phylogenetic and ontogenetic development. J. Anat. 188(Pt 1), 53–58 8655415 PMC1167632

[B67] Miyata T. Kawaguchi A. Okano H. Ogawa M. (2001). Asymmetric inheritance of radial glial fibers by cortical neurons. Neuron 31, 727–741 10.1016/S0896-6273(01)00420-2 11567613

[B68] Miyata T. Kawaguchi A. Saito K. Kawano M. Muto T. Ogawa M. (2004). Asymmetric production of surface-dividing and non-surface-dividing cortical progenitor cells. Development 131, 3133–3145 10.1242/dev.01173 15175243

[B69] Mota B. Herculano-Houzel S. (2012). How the cortex gets its folds: an inside-out, connectivity-driven model for the scaling of mammalian cortical folding. Front. Neuroanat. 6:3 10.3389/fnana.2012.00003 22347170 PMC3270328

[B70] Neil J. J. Shiran S. I. McKinstry R. C. Schefft G. L. Snyder A. Z. Almli C. R. (1998). Normal brain in human newborns: apparent diffusion coefficient and diffusion anisotropy measured by using diffusion tensor MR imaging. Radiology 209, 57–66 9769812 10.1148/radiology.209.1.9769812

[B71] Nimchinsky E. A. Gilissen E. Allman J. M. Perl D. P. Erwin J. M. Hof P. R. (1999). A neuronal morphologic type unique to humans and great apes. Proc. Natl. Acad. Sci. U.S.A. 96, 5268–5273 10.1073/pnas.96.9.5268 10220455 PMC21853

[B72] Noctor S. C. Flint A. C. Weissman T. A. Dammerman R. S. Kriegstein A. R. (2001). Neurons derived from radial glial cells establish radial units in neocortex. Nature 409, 714–720 10.1038/35055553 11217860

[B73] Noctor S. C. Martínez-Cerdeño V. Ivic L. Kriegstein A. R. (2004). Cortical neurons arise in symmetric and asymmetric division zones and migrate through specific phases. Nat. Neurosci. 7, 136–144 14703572 10.1038/nn1172

[B74] Pagel M. (1999). Inferring the historical patterns of biological evolution. Nature 401, 877–884 10.1038/44766 10553904

[B75] Palaniyappan L. Liddle P. F. (2012). Aberrant cortical gyrification in schizophrenia: a surface-based morphometry study. J. Psychiatry Neurosci. 37, 399–406 10.1503/jpn.110119 22640702 PMC3493098

[B76] Panizzon M. S. Fennema-Notestine C. Eyler L. T. Jernigan T. L. Prom-Wormley E. Neale M. (2009). Distinct genetic influences on cortical surface area and cortical thickness. Cereb. Cortex 19, 2728–2735 10.1093/cercor/bhp026 19299253 PMC2758684

[B77] Peters A. Yilmaz E. (1993). Neuronal organization in area 17 of cat visual cortex. Cereb. Cortex 3, 49–68 10.1093/cercor/3.1.49 7679939

[B78] Pillay P. Manger P. R. (2007). Order-specific quantitative patterns of cortical gyrification. Eur. J. Neurosci. 25, 2705–2712 10.1111/j.1460-9568.2007.05524.x 17459107

[B79] Polleux F. Dehay C. Kennedy H. (1997a). The timetable of laminar neurogenesis contributes to the specification of cortical areas in mouse isocortex. J. Comp. Neurol. 385, 95–116 9268119

[B80] Polleux F. Dehay C. Moraillon B. Kennedy H. (1997b). Regulation of neuroblast cell-cycle kinetics plays a crucial role in the generation of unique features of neocortical areas. J. Neurosci. 17, 7763–7783 9315898 10.1523/JNEUROSCI.17-20-07763.1997 PMC6793912

[B81] Preuss T. M. Coleman G. Q. (2002). Human-specific organization of primary visual cortex: alternating compartments of dense cat-301 and calbindin immunoreactivity in layer 4A. Cereb. Cortex 12, 671–691 10.1093/cercor/12.7.671 12050080

[B82] Rakic P. (1988). Specification of cerebral cortical areas. Science 241, 170–176 10.1126/science.3291116 3291116

[B83] Reep R. L. Johnson J. I. Switzer R. C. Welker W. I. (1989). Manatee cerebral cortex: cytoarchitecture of the frontal region in trichechus manatus latirostris. Brain Behav. Evol. 34, 365–386 10.1159/000116523 2611642

[B84] Reep R. L. O'Shea T. J. (1990). Regional brain morphometry and lissencephaly in the sirenia. Brain Behav. Evol. 35, 185–194 2379080 10.1159/000115866

[B85] Régis J. Mangin J.-F. Ochiai T. Frouin V. Riviére D. Cachia A. (2005). “Sulcal root” generic model: a hypothesis to overcome the variability of the human cortex folding patterns. Neurol. Med. Chir. (Tokyo) 45, 1–17 15699615 10.2176/nmc.45.1

[B86] Reillo I. de Juan Romero C. García-Cabezas M. Á. Borrell V. (2011). A role for intermediate radial glia in the tangential expansion of the mammalian cerebral cortex. Cereb. Cortex 21, 1674–1694 21127018 10.1093/cercor/bhq238

[B87] Rilling J. K. Insel T. R. (1999). The primate neocortex in comparative perspective using magnetic resonance imaging. J. Hum. Evol. 37, 191–223 10.1006/jhev.1999.0313 10444351

[B88] Rogers J. Kochunov P. Zilles K. Shelledy W. Lancaster J. Thompson P. (2010). On the genetic architecture of cortical folding and brain volume in primates. Neuroimage 53, 1103–1108 10.1016/j.neuroimage.2010.02.020 20176115 PMC3137430

[B89] Ross M. E. Walsh C. A. (2001). Human brain malformations and their lessons for neuronal migration. Annu. Rev. Neurosci. 24, 1041–1070 10.1146/annurev.neuro.24.1.1041 11520927

[B90] Sawada K. Fukunishi K. Kashima M. Imai N. Saito S. Sakata-Haga H. (2012a). Neuroanatomic and magnetic resonance imaging references for normal development of cerebral sulci of laboratory primate, cynomolgus monkeys (*Macaca fascicularis*). Congnit. Anom. (Kyoto) 52, 16–27 10.1111/j.1741-4520.2011.00352.x 22348780

[B91] Sawada K. Fukunishi K. Kashima M. Saito S. Sakata-Haga H. Aoki I. (2012b). Fetal gyrification in cynomolgus monkeys: a concept of developmental stages of gyrification. Anat. Rec. (Hoboken) 295, 1065–1074 10.1002/ar.22478 22593081

[B92] Sawada K. Sun X.-Z. Fukunishi K. Kashima M. Saito S. Sakata-Haga H. (2010). Ontogenetic pattern of gyrification in fetuses of cynomolgus monkeys. Neuroscience 167, 735–740 10.1016/j.neuroscience.2010.02.045 20219641

[B93] Schindelin J. Arganda-Carreras I. Frise E. Kaynig V. Longair M. Pietzsch T. (2012). Fiji: an open-source platform for biological-image analysis. Nat. Methods 9, 676–682 10.1038/nmeth.2019 22743772 PMC3855844

[B94] Sherwood C. C. Holloway R. L. Gannon P. J. Semendeferi K. Erwin J. M. Zilles K. (2003). Neuroanatomical basis of facial expression in monkeys, apes, and humans. Ann. N.Y. Acad. Sci. 1000, 99–103 10.1196/annals.1280.021 14766625

[B95] Sherwood C. C. Stimpson C. D. Raghanti M. A. Wildman D. E. Uddin M. Grossman L. I. (2006). Evolution of increased glia-neuron ratios in the human frontal cortex. Proc. Natl. Acad. Sci. U.S.A. 103, 13606–13611 10.1073/pnas.0605843103 16938869 PMC1564260

[B96] Shitamukai A. Konno D. Matsuzaki F. (2011). Oblique radial glial divisions in the developing mouse neocortex induce self-renewing progenitors outside the germinal zone that resemble primate outer subventricular zone progenitors. J. Neurosci. 31, 3683–3695 10.1523/JNEUROSCI.4773-10.2011 21389223 PMC6622781

[B97] Smart I. H. McSherry G. M. (1986). Gyrus formation in the cerebral cortex in the ferret. i. description of the external changes. J. Anat. 146, 141–152 3693054 PMC1166530

[B98] Smart I. H. M. Dehay C. Giroud P. Berland M. Kennedy H. (2002). Unique morphological features of the proliferative zones and postmitotic compartments of the neural epithelium giving rise to striate and extrastriate cortex in the monkey. Cereb. Cortex 12, 37–53 10.1093/cercor/12.1.37 11734531 PMC1931430

[B99] Stephan H. Frahm H. Baron G. (1981). New and revised data on volumes of brain structures in insectivores and primates. Folia Primatol. 35, 1–29 10.1159/000155963 7014398

[B100] Stubbs D. DeProto J. Nie K. Englund C. Mahmud I. Hevner R. (2009). Neurovascular congruence during cerebral cortical development. Cereb. Cortex 19 Suppl. 1, i32–i41 19386634 10.1093/cercor/bhp040 PMC2693536

[B101] Takahashi E. Folkerth R. D. Galaburda A. M. Grant P. E. (2012). Emerging cerebral connectivity in the human fetal brain: an MR tractography study. Cereb. Cortex 22, 455–464 10.1093/cercor/bhr126 21670100 PMC3256410

[B102] Taverna E. Huttner W. B. (2010). Neural progenitor nuclei IN motion. Neuron 67, 906–914 10.1016/j.neuron.2010.08.027 20869589

[B103] Toro R. Burnod Y. (2005). A morphogenetic model for the development of cortical convolutions. Cereb. Cortex 15, 1900–1913 10.1093/cercor/bhi068 15758198

[B104] Toro R. Perron M. Pike B. Richer L. Veillette S. Pausova Z. (2008). Brain size and folding of the human cerebral cortex. Cereb. Cortex 18, 2352–2357 10.1093/cercor/bhm261 18267953

[B105] Van Essen D. C. (1997). A tension-based theory of morphogenesis and compact wiring in the central nervous system. Nature 385, 313–318 10.1038/385313a0 9002514

[B106] Wang X. Tsai J.-W. LaMonica B. Kriegstein A. R. (2011). A new subtype of progenitor cell in the mouse embryonic neocortex. Nat. Neurosci. 14, 555–561 10.1038/nn.2807 21478886 PMC3083489

[B107] Winkler A. M. Kochunov P. Blangero J. Almasy L. Zilles K. Fox P. T. (2010). Cortical thickness or grey matter volume? the importance of selecting the phenotype for imaging genetics studies. Neuroimage 53, 1135–1146 10.1016/j.neuroimage.2009.12.028 20006715 PMC2891595

[B108] Xu G. Knutsen A. K. Dikranian K. Kroenke C. D. Bayly P. V. Taber L. A. (2010). Axons pull on the brain, but tension does not drive cortical folding. J. Biomech. Eng. 132, 071013 10.1115/1.4001683 20590291 PMC3170872

[B109] Zilles K. Armstrong E. Schleicher A. Kretschmann H. J. (1988). The human pattern of gyrification in the cerebral cortex. Anat. Embryol. 179, 173–179 10.1007/BF00304699 3232854

[B110] Zilles K. Palomero-Gallagher N. Amunts K. (2013). Development of cortical folding during evolution and ontogeny. Trends Neurosci. 36, 275–284 10.1016/j.tins.2013.01.006 23415112

